# Do the diagnosis-related group payment reforms have a negative impact?—an empirical study from Western China

**DOI:** 10.3389/fpubh.2025.1550480

**Published:** 2025-04-11

**Authors:** Jia-Yi Wang, Meng-En Chen, Xue-Xuan Wei, Xi-Zhu Lu, Yuan-Rui Zhu, Jing-Yu Yang

**Affiliations:** ^1^School of Health Management, Gansu University of Chinese Medicine, Lanzhou, China; ^2^School of Traditional Chinese Medicine, Beijing University of Chinese Medicine, Beijing, China; ^3^School of Public Health, Lanzhou University, Lanzhou, China

**Keywords:** TCM hospitals, DRG, negative effects, hospitalization times, type of medical insurance, westernization

## Abstract

**Background:**

The first Diagnosis-Related Group (DRG) Reimbursement pilots in China, which started in 2019, marked an essential step in cost control and service efficiency in Chinese hospitals, but some adverse effects inevitably emerged during the implementation of DRG in TCM (traditional Chinese medicine) hospitals. This study aims to explore the positive and negative effects of DRG payment reform and provide a reference for the reform of medical insurance payments in countries that retain traditional medicine.

**Methods:**

Longitudinal data from two hospitals, Qingyang City Hospital of TCM and Tianshui City Hospital of TCM, were retrieved from China’s Gansu Health for All Big Data Platform from June 2016 to June 2022, and the policy effects were assessed using the difference-in-differences (DID) method and mediated-effects model.

**Results:**

The DRG reform reduced hospitalization costs, diagnostic costs, drug costs, and nursing costs by 6.5, 4.2, 7.9, and 26.2%, respectively, in the treatment group hospitals (*p* < 0.01), but increased the hospitalization times by 17.5% (*p* < 0.01); there was a “reimbursement bias” for patients with different types of medical insurance. In the treatment group hospitals, the primary beneficiaries of the reform were urban employees’ basic medical insurance patients, whose costs decreased by 4.9% (*p* < 0.01), with a non-significant effect on out-of-pocket payment patients and free medical care patients; the hospitals in the treatment group tended to reduce the use of Chinese medicine unique diagnostic and therapeutic means and increase the proportion of western medicine treatments under the pressure of the supremacy of costs.

**Conclusion:**

The reform of the DRG payment method has positively impacted the cost control of TCM hospitals, but it has also had some adverse effects. This poses a challenge and prompts a thought about how TCM hospitals can maintain their distinctive advantages by optimizing the design of the DRG system at present.

## 1 Introduction

Rising healthcare costs have been an issue that has plagued policymakers and the public around the world for decades (1). Projections show that global health spending will increase from $7.9 trillion in 2017 to $11 trillion in 2030 (2). Between 2002 and 2022, China’s total health expenditure increased from 579 billion CNY to 8,484.6 billion CNY, more than 13 times, and per capita health expenditure also increased from 451 CNY to 6,010 CNY (3, 4). Health costs are growing faster than GDP and have become a heavy financial burden for countries. Forced by financial pressure, countries around the world have adopted DRG to reshape the financing mechanism of hospitals (5–7). DRG, one of the more advanced payment methods recognized today, has gradually become a key way of medical cost control and medical quality management in high-income countries, rapidly spreading worldwide. After seeing the changes and impacts that DRG brought to healthcare costs in a host of developed countries such as the United States, Germany, and Australia, Chinese scholars have also begun to pay attention to DRG since the late 1980s, followed by large-scale research. Many leaders and scholars in China wanted to learn from the West’s advanced DRG cost management methods. In addition to implementing DRG in the massive western hospitals, pilot DRG reforms were simultaneously carried out in TCM hospitals. In 2019, China’s National Bureau of Medical Security designated 30 cities as the key cities for the disease DRG payment reform, which means that starting from the top of the medical insurance payment method, reform has been in full swing nationwide (8). With the continuous deepening and development of China’s medical insurance system, constructing a medical insurance payment mechanism that considers the characteristics of TCM and conforms to the management model of TCM hospitals has become an urgent issue. To this end, the State has successively issued a series of policies and measures, such as the Three-Year Action Plan for the Reform of DRG/DIP Payment Methods and the Guiding Opinions on Medical Insurance Supporting the Inheritance and Innovative Development of Traditional Chinese Medicine, which explicitly require TCM medical institutions to incorporate the use of TCM specialty therapies and Chinese herbal medicines into the DRG grouping and consideration system, increase the coefficients and scores for TCM medical institutions and TCM diseases, and explore the medical insurance payment method that conforms to the characteristics of TCM.

However, some researchers have suggested that DRG may be ineffective and cause problems (9), including outpatient and inpatient cost shifting (10), health professional resistance (11), increased “human ball” patients (select patients) (12), and disincentives to medical technology (13). The implementation of DRG may have unintended negative consequences due to the large number of stakeholders and the complexity of the causal relationships between the various factors involved. China’s unique dual-track healthcare system blends modern Western medicine with TCM. As a government-supported and institutionalized component, TCM occupies an important position in China’s healthcare system, providing nearly 40% of healthcare services (14). Therefore, it is essential to explore the effects of DRG reforms and their potential challenges in China, where both Western and Chinese medicine are emphasized. This paper focuses for the first time on the policy effects of DRG reform from the perspective of adverse effects. It provides valuable references and insights for other countries that retain traditional medicine in their healthcare reform process.

## 2 Literature review and research hypotheses

### 2.1 Literature review

DRG-based payment systems have become the dominant mechanism for hospital reimbursement in many countries. DRG systems in various countries differ significantly in purpose, grouping, coding, and payment mechanisms. Although they are based on the same concept of disease categorization, they often develop different outcomes (15). In the current research on the impact of DRG payment reform on healthcare costs, domestic and international academics have developed three main representative views: the “cost reduction hypothesis,” the “cost shifting hypothesis,” and the “uncertainty hypothesis.” Scholars who support the “cost-reduction argument” argue that DRG can effectively curb the growth of healthcare costs and reduce the provision of unnecessary healthcare services by providing lump-sum payments for each type of illness (16–19). Scholars who support the “cost-shifting theory” argue that under the DRG payment systems, providers may shift cost savings to patients’ out-of-pocket expenses or outpatient services, thereby increasing the financial burden on patients (20–22). Scholars who support the “uncertainty argument” have argued that it is difficult to generalize about the effects of DRG payments due to factors such as the timing of implementation, geographic variations, and the type of disease (23–25). In summary, domestic and international research on DRG payment methodology reform has the following characteristics. First, DRG payment policy design and implementation results vary significantly due to different institutional environments. Second, most of the current research on the effectiveness of DRG payment method reform has focused on the level of cost changes, with few in-depth and systematic studies of negative effects.

One of the innovations of this paper is that the theoretical world about the pilot reform of DRG mainly focuses on Western hospitals. This study selects China’s first batch of pilot hospitals for the reform of the DRG payment method—Qingyang City Hospital of TCM in Gansu Province as the object of the study and launches the survey from the brand-new angle of the implementation of DRG in Chinese medicine-type hospitals. Another innovative point of this paper is that the empirical studies on the DRG pilot reform at home and abroad mainly focus on the reform’s effect on hospital cost control, but there are few empirical studies on its negative impact. In addition to studying the cost-control effect of DRG, this paper also finds some negative impacts accompanying the reform.

### 2.2 Research hypotheses

#### 2.2.1 DRG payment reform impacts hospitalization costs in TCM hospitals

The DRG payment method has a significant cost-control effect on Western hospitals. It reduces hospitalization costs in TCM hospitals thanks to its precise cost-control mechanism and strategy of incentivizing hospitals to improve efficiency. Under the DRG model, medical insurance organizations set a fixed “packaged price” for specific disease groups and bill hospitals accordingly. It means that the hospital must complete the patient’s treatment within the predetermined cost range, or else the hospital will have to pay for any costs that exceed the standard. Therefore, TCM hospitals and general hospitals with Western medicine as the primary treatment need to control costs as much as possible by guaranteeing treatment effects. Based on the above analysis, this paper proposes the following hypothesis:

*H*1: DRG payment reform reduces hospitalization costs in TCM hospitals.

#### 2.2.2 DRG payment reform affects patient hospitalization times

From the initial design of DRG, the billing unit of DRG payment is “one hospitalization,” and the medical insurance department adopts the management method of total prepayment and disease payment. Although this billing method strengthens providers’ awareness of controlling the service components in a single hospitalization, it also enhances the incentives for hospitals to break down the hospitalization (26). Healthcare institutions may reduce costs by breaking down hospitalization or high-value charges to avoid actual costs exceeding the packaged amount or to obtain more balances. To avoid exceeding the packaged amount or obtain a larger balance, providers may reduce costs by breaking up hospitalizations or high charges, which means breaking up a complete hospitalization or surgical procedure to get two or more DRG settlements. The cost containment brought about by DRG and the quality of care has strengthened the “incentive to break up hospitalizations.” The” motivation to dismantle hospitalization” is continuously reinforced by the combination of cost control brought by DRG and quality management. In summary, this paper proposes the following hypothesis:

*H*2: DRG payment reforms decompose the hospitalization times.

#### 2.2.3 DRG payment reform affects the structure of Chinese and Western medical services in TCM hospitals

China’s current DRG payment system and evaluation system are based on Western medicine diagnosis. There are problems, such as the need for more unity between the DRG system and TCM diagnosis and treatment and a compensation mechanism for TCM characteristics. When patients are hospitalized, due to the slower onset of effect of proprietary Chinese medicines compared with Western medicines, TCM diagnosis and treatment adopts appropriate TCM technology to regulate patients’ chronic diseases, which significantly increases the hospitalization times of the patients, resulting in the need for hospitals to bear more operational costs such as workforce costs. However, under the DRG payment system, hospitals’ income is only linked to the types of diseases; the cost increase will not be subsidized more, and hospitals will not receive more medical compensation for the rise in the content of services (27).TCM hospitals will gradually use Western medicines to treat patients. The proportion of Western medicines will be higher and higher, and there is a “shift from Chinese to Western” in medical costs (28). Therefore, this paper proposes the following hypothesis:

*H*3: The reform of DRG payment methodology has driven a shift in the cost structure of Chinese and Western medical care by reducing the proportion of Chinese medicine costs, and the phenomenon of "de-Chinese medicine" has emerged in TCM hospitals.

#### 2.2.4 DRG payment reform affects the cost-control effectiveness of different medical insurance types

The differences in the cost-control effect of DRG payment reform on different types of medical insurance mainly stem from the differences in payment standards, coverage, and reimbursement ratios of different types of medical insurance (such as urban employees’ basic medical insurance and urban residents’ basic medical insurance). Employees’ basic medical insurance covers mainly urban workers, with a relatively high level of protection, usually enjoying a higher reimbursement rate and more lenient payment standards, which makes it easier for hospitals to control costs and obtain reasonable compensation under the DRG framework. In contrast, other types of medical insurance, such as urban residents’ basic medical insurance, have a relatively low level of protection, with low reimbursement rates and stricter payment standards, which makes them benefit much less than the employees’ basic medical insurance in terms of cost control. Accordingly, this paper proposes the following hypothesis:

*H*4: DRG payment reforms have a "reimbursement bias" across different types of medical insurance, and urban employees’ basic medical insurance patients are the primary beneficiaries.

## 3 Materials and methods

### 3.1 Data sources and sample selection

The data in this paper come from the National Health and Wellness Big Data Platform (Government non-full disclosure database) of the Health Commission of Gansu Province, China. The data content mainly involves the information on the front page of the medical records of TCM hospitals, including patient information and longitudinal data on various types of hospitalization costs. In this paper, two tertiary-level TCM hospitals in southeastern Gansu Province—Qingyang City TCM Hospital and Tianshui City TCM Hospital—were selected as the treatment and control groups, respectively; the former is a national DRG reform pilot hospital, and the latter has been following the fee-for-service (FFS) method during the same period. Taking the 2019 reforms as the time point and considering the resident population, economic development, and patient characteristics of the two regions, the two hospitals were found to be in similar overall situations. In terms of population size, Qingyang City has a population of 2,278,800, and Tianshui City has a population of 3,368,900, which are similar in size; in terms of economic level, both cities are located in the less developed region of Gansu province, and the per capita disposable incomes of the urban and rural residents in Qingyang city are 20,023 CNY and 8,897 CNY, respectively, while those of Tianshui city are 156,361 CNY and 9,519 CNY, respectively, which are basically at the same level of economic development. In addition, both cities are located in the southeastern part of Gansu Province, with a straight-line distance of about 300 km, with similar dietary habits and endemic disease profiles of the residents. The two selected hospitals are the largest tertiary Chinese medicine hospitals in the area, which not only ensures the consistency of the level of medical services but also implies that the socio-economic backgrounds and health needs of the patients who visit the hospitals have a high degree of similarity. Therefore, we extracted the two hospitals from June 2016 to June 2022 for cleaning and screening of medical record home page data of hospitalized patients. The study limited the samples according to the following criteria: ① missing information on the medical record home page could not be based on the supplement or logical error; ② the number of days of hospitalization was less than 1 or greater than 150 days; ③ the cases with hospitalization costs and the hospitalization times were 0. Finally, the total number of cases included in Qingyang City Hospital of TCM was 111,368 cases, and the total number of cases included in Tianshui City Hospital of TCM was 90,211 cases.

### 3.2 Research methodology and modeling

#### 3.2.1 DID model

Difference-in-differences (DID) is a quasi-experimental methodology well suited to analyzing the impact of policies using longitudinal data (29, 30). The net effect of an intervention is estimated by comparing the difference in change before and after the intervention between the treatment group (the group that received the intervention) and the control group (the group that did not receive the intervention). The simplest form of the DID model is the two-group, two-period design, in which the study sample is divided into two groups: the treatment group and the control group, represented by a dummy variable. Also, the study time is divided into two periods: pre-intervention and post-intervention, represented by another dummy variable. In this simple DID, the treatment variable is the product of these two dummy variables. This paper is based on China’s 2019 DRG payment reform as a natural experiment. Qingyang City Hospital of TCM, exposed to the DRG pilot, is selected as the treatment group. Tianshui City Hospital of TCM, which was not exposed to the DRG pilot, was selected as the control group. The time change in hospitalization cost in the two hospitals is compared. The net effect of the DRG reform policy was assessed using the DID method, and the following initial model was constructed:


(1a) $$Lny_{\mathrm{it}}=\beta_{0}+\beta_{1}\mathrm{Trea}t_{i}\times Post_{t}+\beta_{2}\mathrm{Contro}l_{\mathrm{it}}+\delta_{i}+\mu_{t}+\varepsilon_{\mathrm{it}}$$

In Equation 1a, *Lny_it_* denotes the logarithmic form of each type of hospitalization cost; i denotes inpatient, t denotes time; *Treat_i_* denotes binary grouping variable, taking 1 for the treatment group and 0 for the control group; *Post_t_* denotes the time dummy variable, taking 1 after the policy intervention and 0 otherwise; *Control_it_* denotes a set of control variables; $\delta_{i}$ denotes individual-fixed effects; $\mu_{t}$ denotes time-fixed effects, $\varepsilon_{\mathrm{it}}$ is a random error term; $\beta_{0}$ denotes a constant term; the coefficients $\beta_{1}$ in the model before the interaction term $\mathrm{Trea}t_{i}\times Post_{t}$ is the policy effects of interest in this paper, reflecting the effects of the interaction term is 1 and only 1 (31).

A vital prerequisite for using DID is that it meets the “common trend” assumption. This means that the trend in the hospitalization cost for patients in DRG payment reform and non-reform hospitals is roughly convergent without policy intervention (32). In this paper, we refer to D’Haultfoeuille et al.’s methodology and test it using event study methodology, the results of which are presented later (33).

#### 3.2.2 Mediating effect model

Mediating effects are used to describe the process by which one variable (independent variable X) indirectly influences a third variable (dependent variable Y) through another variable (mediating variable M). The mediating effect refers to the fact that the effect of X on Y is realized through M, which means that M is a function of X and Y is a function of M (Y-M-X). Considering the effect of the independent variable X on the dependent variable Y, M is said to be the mediating variable if X affects the variable Y through M. In order to test whether the proportion of TCM expenses can play a mediating effect in the process of DRG payment reform, this paper refers to the classic mediating effect model of Wen Z et al. and constructs the model as follows (34):


(1b) $$Lny_{\mathrm{it}}=\beta_{0}+\beta_{1}\mathrm{Trea}t_{i}\times Post_{t}+\beta_{2}\mathrm{Contro}l_{\mathrm{it}}+\delta_{i}+\mu_{t}+\varepsilon_{\mathrm{it}}$$

(2) $$\mathrm{RatioTC}M_{\mathrm{it}}=\alpha_{0}+\alpha_{1}\mathrm{Trea}t_{i}\times Post_{t}+\alpha_{2}\mathrm{Contro}l_{\mathrm{it}}+\delta_{i}+\mu_{t}+\varepsilon_{\mathrm{it}}$$

(3) $$\begin{array}{l} Lny_{\mathrm{it}}=\gamma_{0}+\gamma_{1}\mathrm{Trea}t_{i}\times Post_{t}+\gamma_{2}\mathrm{RatioTC}M_{\mathrm{it}}\\ +\gamma_{3}\mathrm{Contro}l_{\mathrm{it}}+\delta_{i}+\mu_{t}+\varepsilon_{\mathrm{it}} \end{array}$$

Equation 1b is the same as in the previous section, and in Equations 2, 3, the meaning of each variable is the same as in Equation 1b. Among them, $\alpha_{0}$ and $\gamma_{0}$ represent constant terms, and *RatioTCM_it_* is the ratio of the TCM cost of hospitalized patients to the total cost of TCM and Western medicine, reflecting the degree of TCM use by patients. The coefficient $\beta_{1}$ on $\mathrm{Trea}t_{i}\times Post_{t}$ in Equation 1b captures the overall effect of the impact of DRG reform on patient inpatient costs, and the coefficient $\alpha_{1}$ on the interaction term in Equation 2 represents the impact of DRG reform on the proportion of costs in the TCM. Equation 3 adds *RatioTCM_it_* to Equation 1b, at which point the coefficient $\gamma_{1}$ before $\mathrm{Trea}t_{i}\times Post_{t}$ represents the direct impact of the DRG reform on hospitalization costs. According to the mediation effect test procedure, if $\alpha_{1}$ in Equation 2 and $\gamma_{1}$ in Equation 3 are both significantly negative, and $\gamma_{2}$ in Equation 3 is also significantly negative, which means that the proportion of TCM costs plays a partly mediating role. This suggests that the hospitals in the treatment group have achieved the effect of controlling the costs by reducing the proportion of TCM costs, which will be followed up by this test.

### 3.3 Variables design variables

Dependent Variables. Various types of hospitalization costs include the patient’s total hospitalization costs *Lny1*, diagnostic costs *Lny2*, drug costs *Lny3*, and nursing costs *Lny4*.

Key variables. *Treat_i_* represents the policy dummy variable, taking 1 if the patient belongs to the treatment group hospitals and 0 otherwise. *Post_t_* represents the time dummy variable, the DRG policy treatment point is October 2019, and patients discharged between June 2016–September 2019 are regarded as pre-reform and assigned the value of 0; patients discharged between October 2019–June 2022 are regarded as post-reform and assigned a value of 1. $\mathrm{Trea}t_{i}\times Post_{t}$ serves as the cross-multiplier of the policy dummy variable and the time dummy variable, whose coefficients, which are also the focus of this study, can reflect the net effect of the DRG policy.

Control Variables. *Control_it_* is the control variable of this paper. This paper draws on existing literature (35, 36) and introduces the following control variables: from the perspective of patients’ characteristics, the control variables “age ““gender ““marital status ““occupation “and “types of insurance “were added; from the comprehensive characteristics of hospitals, the control variables “clinical pathway ““types of treatment ““use of TCM therapeutic equipment ““use of TCM treatment techniques ““surgeries and operations ““number of hospital beds “and “case mix index (CMI) value “are added. In addition, given that the new crown outbreak in China in 2019 impacted the total number of hospitalized cases and hospitalization costs in some regions, this study also included the “outbreak of COVID-19″ as a control variable in the model to exclude its confounding effect.

## 4 Results

### 4.1 Descriptive analysis

The results of descriptive statistics are shown in Table 1. The mean value of hospitalization cost y1 is 7514.493, which is greater than the median of 6487.038, and it is a right-skewed distribution affected by the great value. The maximum value is 224875.8, and the minimum value is 5, indicating heterogeneity in hospitalization cost. The logarithm is taken to be Lny1, and the treatment of y2-y4 is the same. To circumvent the issue of multicollinearity, this study employed the variance inflation factor (VIF) test for all variables. The results of this analysis demonstrate that no multicollinearity exists between the variables.

**Table 1:** Regression model variable settings and descriptive statistics results.

Variable	Variable definitions and descriptions	N	Mean	SD	Median	Min	Max
Key variables							
Treat×Post	–						
Treat	–	–	–	–	–	–	–
Post	–	–	–	–	–	–	–
Dependent variables							
y1	Hospitalization Cost (CNY¥)	201,579	7514.493	6487.038	6372.24	5	224875.8
y2	Diagnosis Cost (CNY¥)	201,579	1927.846	1227.978	1762.00	0	48602.0
y3	Medicine Cost (CNY¥)	201,579	2493.308	2482.983	1962.03	0	101005.5
y4	Nursing Cost (CNY¥)	201,579	1225.142	1772.919	669.80	0	48000.0
Control variables							
Age	Respondent’s Age	201,579	51.446	22.173	55.00	0	99.0
Gender	Male = 1	100,932	–	–	–	1	2
	Female = 2	100,647					
Marital Status	Married = 1	163,228	–	–	–	1	2
	Not married = 2	38,351					
Occupation	Occupation of Respondents	201,579	–	–	–	1	9
Clinical Pathway	Traditional Chinese Medicine = 1	45,770	–	–	–	1	3
	Western Medicine = 2	6,661	–	–	–	1	3
	Other = 3	149,148					
Types of Insurance	Urban Employees’ Basic Medical Insurance = 1	27,674	–	–	–	1	5
	Urban Residents’ Basic Medical Insurance = 2	19,783	–	–	–	1	5
	Out-of-Pocket Payment =3	6,896	–	–	–	1	5
	Free Medical Care =4	185	–	–	–	1	5
	Other = 5	147,041	–	–	–	1	5
Types of Treatment	TCM Treatment = 1	150,474	–	–	–	1	3
	Western Medical Treatment = 2	2,735					
	TCM and Western Medical Treatment = 3	48,370					
Use of TCM therapeutic equipment	Yes = 1	177,806	–	–	–	1	2
	No = 2	23,773					
Use of TCM treatment techniques	Yes = 1	161,521	–	–	–	1	2
	No = 2	40,058					
Surgery and procedures	Yes = 1	11,191	–	–	–	1	2
	No = 2	190,388					
Outbreak of COVID-19^*^	Yes = 1	97,654	–	–	–	1	2
	No = 2	103,925					
Number of hospital beds	Actual number of hospital beds opened	653	653	103.346	730	500	730
CMI	Case Mix Index	201,579	1.232	0.1454	1.35	1.03	1.35

*Due to the temporal differences between the two places affected by the outbreak, the time of the first new crown case appearing in each of the two cities was chosen as the start of the outbreak. The first new crown case appeared in Tianshui City on January 28, 2020, and in Qingyang City on February 10, 2020.

### 4.2 Did estimates

Table 2 reports the regression results of the benchmark regressions. Columns (1) (3) (5) (7) are the estimation results without the inclusion of control variables and without controlling for individual-fixed and time-fixed effects, while columns (2) (4) (6) (8) are the estimation results with the inclusion of control variables and controlling for fixed effects. In column (1) (2), the coefficients of Treat×Post are −0.114 and − 0.065 before and after the inclusion of control variables, both significant at the 1% level, indicating that the hospitalization cost Lny1 of patients in hospitals in the treatment group decreases after the DRG reform. Hypothesis 1 of this paper is verified. Similarly, the Treat×Post coefficients in column (3)-column (6) are all negative, and the DRG payment reform has a better cost-control effect on inpatient diagnostic cost lny2 and drug cost lny3, which decreased by 4.2 and 7.9%, respectively, regardless of the inclusion of control variables and fixed effects (*p* < 0.01). From columns (7) and (8), it can be concluded that the coefficient of Treat×Post is insignificant (*p* > 0.05) before adding control variables and controlling for fixed effects. After adding control variables and controlling for fixed effects, the DRG reform significantly impacts the nursing cost lny4 in the treatment group of hospitals, with a decrease of nearly 26.2% (*p* < 0.01).

**Table 2:** Baseline regression.

Variables	Lny1		Lny2		Lny3		Lny4	
	(1)	(2)	(3)	(4)	(5)	(6)	(7)	(8)
Treat×Post	−0.114 ^***^	−0.065^***^	0.025 ^***^	−0.042^***^	−0.367^***^	−0.079^***^	0.011	−0.262^***^
	(−33.05)	(−10.02)	(6.61)	(−5.53)	(−73.52)	(−6.94)	(1.49)	(−11.94)
Gender		−0.025^***^		−0.091^***^		−0.030^***^		0.080^***^
		(−7.61)		(−22.95)		(−5.21)		(7.83)
Age		0.014^***^		0.022^***^		0.021^***^		0.000^*^
		(167.06)		(195.71)		(148.58)		(1.66)
Marital status		0.001^***^		0.001^***^		0.002^***^		−0.000
		(7.92)		(6.81)		(9.37)		(−0.84)
Occupation		0.000		−0.003^***^		0.001^***^		−0.002^***^
		(0.96)		(−37.39)		(12.05)		(−12.56)
Types of Insurance		−0.015^***^		−0.027^***^		−0.009^***^		−0.019^***^
		(−9.67)		(−9.71)		(−6.56)		(−6.44)
Types of Treatment		−0.087^***^		−0.113^***^		−0.075^***^		−0.296^***^
		(−21.44)		(−23.41)		(−10.31)		(−25.02)
Clinical Pathway		−0.015^***^		−0.009^***^		−0.001		−0.178^***^
		(−7.99)		(−4.03)		(−0.29)		(−29.89)
Use of TCM therapeutic equipment		0.009		0.000		0.049^***^		−0.455^***^
		(1.34)		(0.05)		(4.23)		(−24.64)
Use of TCM treatment techniques		−0.160^***^		−0.030^*^		−0.283^***^		−0.379^***^
		(−11.10)		(−1.80)		(−11.54)		(−8.34)
Surgery and procedures		0.390^***^		−0.020^***^		0.036^***^		−0.282^***^
		(61.76)		(−2.89)		(3.30)		(−15.34)
Outbreak of COVID-19		−0.065^***^		0.013^*^		−0.186^***^		0.303^***^
		(−10.70)		(1.76)		(−16.93)		(14.42)
Constant	8.731 ^***^	8.308^***^		6.742^***^	7.538^***^	6.700^***^	6.580^***^	8.215^***^
	(5082.31)	(485.84)		(329.69)	(3042.98)	(233.53)	(1925.87)	(157.69)
Individual-fixed effect	×	✓	×	✓	×	✓	×	✓
Time-fixed effect	×	✓	×	✓	×	✓	×	✓
N	201,579	89,671	201,132	89,533	200,413	89,135	178,690	77,501
R-squared	0.0054	0.3611	0.0002	0.4698	0.0263	0.2587	0.0000	0.0685

1. ***p < 0.01, **p < 0.05, *p < 0.1; 2. *t*-values in parentheses, models are with robust standard errors.

### 4.3 Robustness test results

#### 4.3.1 Parallel trend test

**Figure 1:**
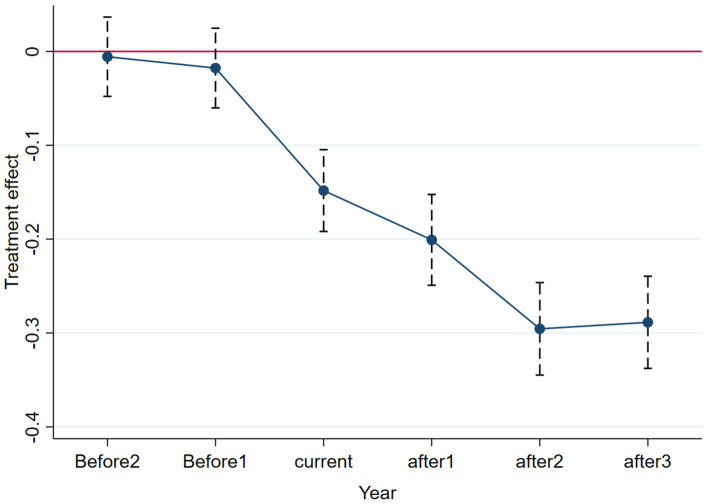
Parallel trend test.

The use of the DID model presupposes that the data satisfy the parallel trend assumption and that the experimental and control groups should have had the same trend before the policy was implemented; it shows that there is no significant variability in the trend of patient inpatient costs over time between the treatment and control hospitals in the absence of the DRG payment reform (37). Figure 1 is a parallel trend plot reporting the estimated coefficients of the core explanatory variable Treat×Post and its 95% confidence interval. To avoid the problem of covariance, we refer to the literature of existing scholars and select the base period (2016) as the benchmark group to remove it (38). The parallel trend graphs show the test results for 2 years before the reform and 3 years after the reform started. Before the implementation of the policy, the confidence interval covers 0, and the estimated coefficients are insignificant, so there is no significant difference between the control and treatment groups before the implementation of the DRG payment policy. After the implementation of DRG payment, the estimated coefficient of the interaction term is significantly non-zero, and the DRG payment method effectively reduces the hospitalization cost of the hospitals in the treatment group and shows a decreasing trend year by year, passing the parallel trend test.

#### 4.3.2 Placebo test

**Figure 2:**
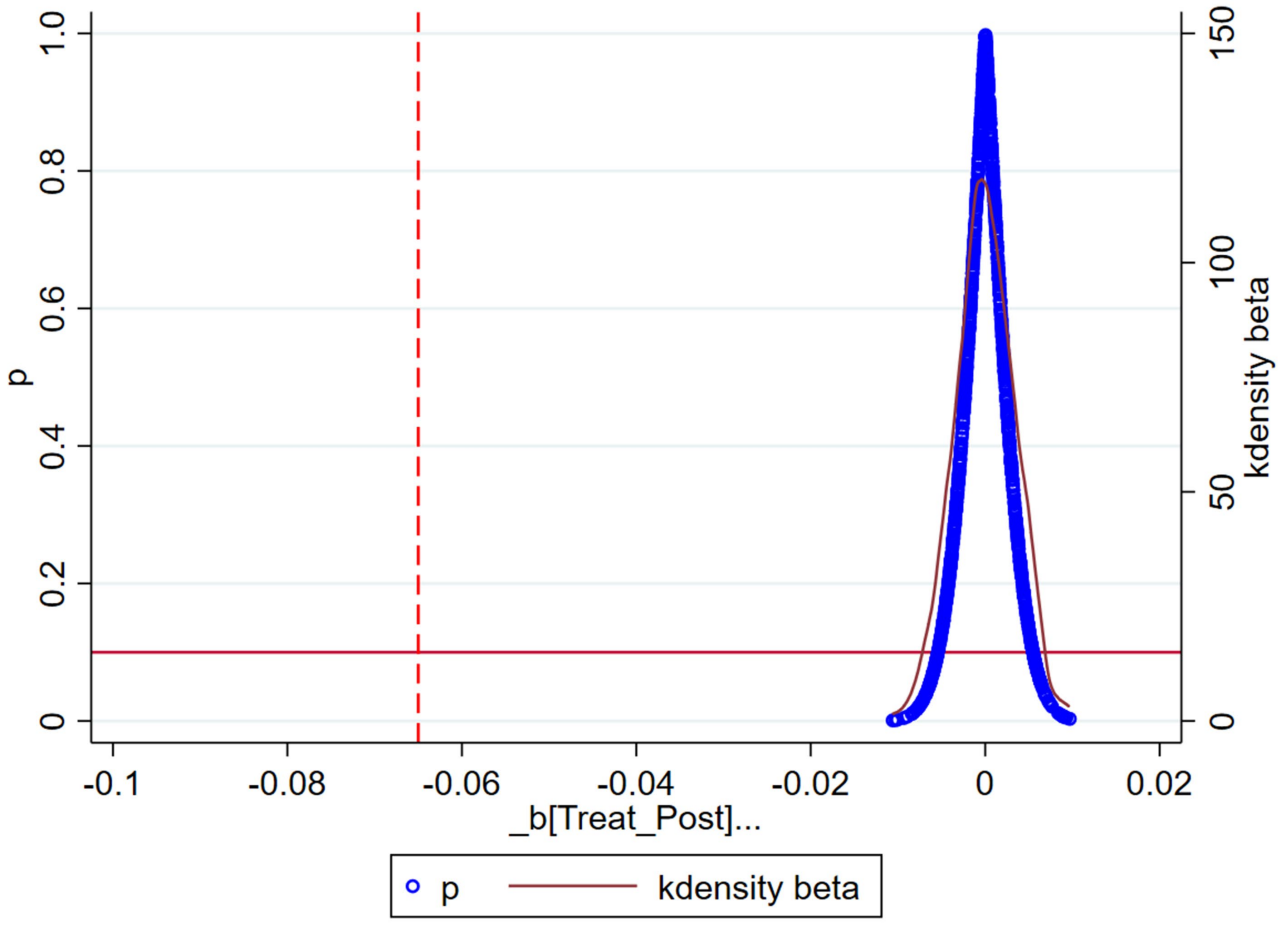
Placebo test.

To avoid the baseline regression results being affected by unobservable omitted variables, a fictitious experimental group conducts a placebo test (39). In this paper, the coefficient estimates of the effect of the DRG payment method on hospitalization costs are obtained by taking 500 cases from the entire sample as a dummy experimental group and the remaining patients as a dummy control group and regressing them. The above process was repeated 1,000 times to obtain 500 regression coefficients. Figure 2 depicts the probability distribution of the estimated coefficients of the interaction term Treat×Post under conducting 500 random samples. As shown in Figure 2, the estimated coefficient values obtained based on random sampling are distributed around 0, while the estimated coefficient of the benchmark regression, −0.065, lies outside the coefficient distribution and is a small probability event. Therefore, it can be ruled out that the results of the benchmark regression in this paper are due to unobservable factors, and the results of the negative impact of the DRG payment reform on the reduction of hospital inpatient costs in the treatment group are robust.

### 4.4 Further findings

#### 4.4.1 Negative effect 1: higher hospitalization times

While DRG reforms have had significant success in reducing hospitalization costs, they have also had the negative consequences of decomposed hospitalizations. Decomposed hospitalization refers to hospital efforts to circumvent DRG cost constraints by breaking down what should be a single inpatient course of treatment into multiple, shorter stays, which can occur when hospitals seek to control spending overruns in a single group or to capture more DRG payments. Based on the platform data, we selected the same patient’s cumulative hospitalization times in a year as a statistical indicator to study patient readmissions. As shown in Table 3, before and after the implementation of the reform, the average hospitalization times for patients in non-DRG hospitals increased by 0.075. In contrast, the hospitalization times for patients in DRG hospitals increased by 0.157. Relative to the control group, hospitals in the treatment group experienced a significant increase in hospitalization times after the DRG policy intervention.

**Table 3:** Hospitalization times of patients.

Variables	June 2016–September 2019 (Pre-reform)	October 2019–June 2022 (Post-reform)	Difference (the result of subtraction)
Non-DRG hospital	1.041	1.116	0.075
DRG hospitals	1.652	1.809	0.157

To further investigate whether there was a decomposition of hospitalization in the treatment group hospitals, we used the hospitalization times of patients as an explanatory variable, and the core explanatory variables and control variables were set as in the previous section for further regression analyses. The regression results in Table 4 show that the coefficients of the interaction terms are 0.556 and 0.175 (*p* < 0.01), regardless of whether control variables and fixed effects are included or not, and this result indicates that the DRG payment reform significantly increases the hospitalization times in DRG hospitals, which further verifies the negative consequences of the existence of decomposed hospitalizations in hospitals implementing the DRG reform. This is consistent with the findings of some scholars (40, 41). Hypothesis 2 of this paper is verified.

**Table 4:** Regression results of hospitalization times.

Variables	Dependent variables: Hospitalization times	
	(1)	(2)
Treat×Post	0.556 ^***^	0.175^***^
	(68.70)	(6.91)
Constant	1.253^***^	1.636 ^***^
	(328.74)	(29.19)
Control variables	×	✓
Individual-fixed effect	×	✓
Time-fixed effect	×	✓
N	170,427	77,914
R-squared	0.027	0.039

1. ***p < 0.01, **p < 0.05, *p < 0.1; 2. *t*-values in parentheses, models are with robust standard errors.

#### 4.4.2 Negative effect 2: westernization of TCM hospitals

Apart from breaking down the hospitalization times of patients, the DRG reform has led to changes in the cost structure of Chinese and Western medicines in the hospitals of the treatment group, with the proportion of Chinese medicine decreasing and that of Western medicine increasing. This will lead to the loss of Chinese medicine’s original characteristics and the advantages of Chinese medicine hospitals, and the gradual “Westernization” of Chinese medicine. In this study, *RatioTCM* is used as the mediating variable for the proportion of Chinese medicine costs for inpatients, and the regression results of the mediating effect are shown in Table 5. The coefficient of Treat×Post in column (1) is −0.065, indicating that the DRG reform pushes down the inpatient costs of patients in the hospitals of the treatment group, and the total effect of the impact of the DRG reform on the inpatient costs of patients is −0.065; the coefficient of Treat×Post in column (2) is −0.033, indicating that the DRG reform reduces the share of costs in the TCM; the coefficient of Treat×Post in column (3) is −0.064 after the addition of the mediating variable *RatioTCM*, indicating that the direct effect of the DRG reform on the impact of inpatient costs is −0.064 The Sobel-Goodman test and Bootstrap test (see Supplementary material for specific test results) confirm that a partial mediation effect exists, and the proportion of the mediation effect is 6.661%; this indicates that the DRG reform reduces inpatient hospitalization costs by lowering the proportion of costs in the TCM. Hypothesis 3 of this paper is verified.

**Table 5:** The mediating effect test of the proportion of TCM costs.

Variables	Dependent variables		
	Lny1	Ratio TCM	Lny1
	(1)	(2)	(3)
RatioTCM			−0.037^***^
			(−5.05)
Treat×Post	−0.065^***^	−0.033^***^	−0.064^***^
	(−10.02)	(−10.47)	(−9.99)
Constant	8.308^***^	1.117^***^	8.315^***^
	(485.84)	(154.20)	(453.89)
Control variables	✓	✓	✓
Individual-fixed effect	✓	✓	✓
Time-fixed effect	✓	✓	✓
N	89,671	89,352	89,352
R-squared	0.3611	0.2255	0.3669

1. ***p < 0.01, **p < 0.05, *p < 0.1; 2. *t*-values in parentheses, models are with robust standard errors.

#### 4.4.3 Negative effect 3: reimbursement bias

In addition to the two negative effects described above, the DRG payment reform has a third negative effect: it has a different cost-control effect on patients with different types of health insurance, and there is a problem of “reimbursement bias.” The results of the heterogeneity analysis confirmed this study. We used *Lny1*, the hospitalization cost of patients with varying types of medical insurance, as the dependent variable, and the regression results are shown in Table 6. The coefficients on Treat×Post in columns (1) and (2) are all negative (*p* < 0.01), indicating that the DRG payment reform reduced hospitalization costs by 4.9% (p < 0.01) for urban workers’ basic health insurance patients and 3.8% (p < 0.01) for urban residents’ basic health insurance patients. The Treat×Post coefficients in columns (3) and (4) are not significant (*p* > 0.05) for out-of-pocket payment and free medical care patients. This may be due to the lack of bargaining power of out-of-pocket patients, and the “the government bears the cost “nature of the free medical care system, which makes it difficult for DRG reforms to effectively control costs and leads to a lack of awareness of savings among providers and patients. The above conclusion proves a significant difference in the impact of DRG reform on the hospitalization costs of patients with different types of insurance, and Hypothesis 4 of this paper is verified.

**Table 6:** Heterogeneity analysis of hospitalization costs for patients with different types of insurance coverage.

Variables	Dependent variables: Lny1			
	(1) Urban employees’ basic medical insurance	(2) Urban residents’ basic medical insurance	(3) Out-of-pocket payment	(4) Free medical care
Treat×Post	−0.049^***^	−0.038^***^	0.030	−0.346
	(−4.58)	(−3.12)	(1.17)	(−1.68)
Constant	8.716^***^	8.164^***^	8.243^***^	7.600^***^
	(288.53)	(242.52)	(123.06)	(22.17)
Control variables	✓	✓	✓	✓
Individual-fixed effect	✓	✓	✓	✓
Time-fixed effect	✓	✓	✓	✓
N	27,505	19,691	6,853	182
R-squared	0.0929	0.4081	0.3867	0.5414

1. ***p < 0.01, **p < 0.05, *p < 0.1; 2. *t*-values in parentheses, models are with robust standard errors.

## 5 Discussion

DRG reform is currently a key area of healthcare reform in the world. Given the indispensable role of TCM in China’s healthcare system, TCM is also a focus of DRG reform. China’s implementation of DRG reform in TCM hospitals has had a series of impacts (42). This paper is an empirical investigation of the first tertiary-level TCM hospital in western China that implemented DRG payment reform. The DRG model implemented in TCM healthcare organizations has demonstrated significant cost-control effects while exposing potential problems. This study examines the impact of this reform in depth.

Benchmark regression results show a downward trend in hospitalization costs in the treatment group of hospitals with DRG reforms, indicating that DRG payment reforms have better cost-control effects than FFS payment methods. This is consistent with L. Y’s 2023 study about L City in China (43). As a system design originating in the United States and mainly used for reimbursement methods and inpatient costs in Western hospitals, DRG payment reform in Gansu Province in China shows that transplanting DRG to Chinese TCM hospitals can still achieve significant results in controlling inpatient costs. However, it also has some negative effects, and TCM hospitals worldwide have experienced the phenomenon of “not adapting to the local conditions” in the reform process (44).

The regression results revealed that hospitals in the treatment group experienced a significant increase in hospitalization times after the DRG policy intervention, and the phenomenon of decomposing hospitalizations may exist. The hospitals in the treatment group re-admitted patients for the same disease or the same symptoms within a short period, breaking down the treatment process that should be completed in one hospitalization for patients into two or more. The above behavior was a strategy the treatment group hospitals adopted to cope with the strict cost control requirements. Because disaggregated admissions are an act of “self-defense” by hospitals in response to medicare payment policies (45). A 2012 study by Hamada H et al. also showed that introducing the DRG system in Japan in 2003 increased hospital readmission rates (46). Interestingly, we also found that hospital CMIs in the treatment group increased after the DRG reform, but the associated hospitalization costs decreased somewhat. A rise in CMI usually indicates an increase in the case’s complexity, which should be accompanied by increased resource consumption (e.g., higher costs). However, the hospitalization cost in the treatment group declined. This contradiction suggests that hospitals may achieve “efficiency gains at the data level” (higher CMI and lower total costs) through strategies such as “strategic upcoding” and “disaggregated hospitalization.” It may hide the risks of increasing patient burden and wasting health insurance funds.

We found a second negative effect of the DRG reform through a mediation effect model: the implementation of the DRG led to fewer TCM services being provided by the treatment group hospitals, resulting in a shift of some of the healthcare costs from Western to Chinese. Facing the fixed payment standard of the DRG payment method, Chinese hospitals are caught up in the consideration of cost control, and the DRG payment method is only related to the grouping of cases. Hospitals will not receive more medical compensation for the increased service content, so TCM hospitals will reduce the use of Chinese medicine treatments to reduce costs (47). The characteristic advantages of Chinese medicine are the foundation for the further development of TCM hospitals and the unique core competitiveness of TCM hospitals (48). The unique advantages of Chinese medicine of “simplicity, inexpensiveness, convenience, and testing” make Chinese medicine in inpatient hospitalization services have advantages that cannot be compared with Western medicine, but Chinese medicine treatment has the disadvantage of long treatment period, which is also the root reason of “de-Chinese medicalization “in TCM hospital.

The third negative effect of DRG reform on TCM hospitals is analyzed by the heterogeneity of the types of medical insurance, which results in the differentiation of the fee-control effects of DRG payment reform across different types of medical insurance, leading to fee-control discrimination in the implementation of a system that is supposed to be universally beneficial. Patients cannot enjoy the benefits of medical insurance and the dividends of the DRG reform equitably. DRG has a “reimbursement preference” for patients with varying types of medical insurance. In this study, the decline in hospitalization costs for employees’ medical insurance patients in the policy group hospitals was higher than that for urban residents’ medical insurance patients. Employees’ medical insurance patients were the primary beneficiaries of DRG reform, which is consistent with the conclusions of current scholars (49, 50). In addition, due to the differences in the insured groups of different types of insurance, urban residents’ basic medical insurance and out-of-pocket payment patients are primarily farmers and people with no fixed work units, whose economic level is limited and whose health risks are higher than those of patients with urban employees’ basic medical insurance and free medical care. As a result, the severity of illnesses among patients attending hospitals varies considerably, further exacerbating the differences in healthcare costs among patients with different types of medical insurance.

## 6 Limitations

There are some limitations of this study. First, because this paper selected all inpatient cases from the TCM hospital from June 2016 to June 2022, the study cases and study time were limited. With the further advancement of the DRG payment system, we can choose more samples and time for the study. Second, due to data availability, this study relied on front-page medical records and focused on changes in hospitalization costs, and lacked valid assessment of clinical efficacy metrics, medical and surgical department data. This limitation is mainly due to the underdeveloped case system in TCM hospitals, which will improve with future case information collection system updates. Third, the subjects of this study were selected from the first DRG pilot TCM-type hospitals in China as the treatment group, and nearby non-pilot TCM-type hospitals were selected as the control group. No comparisons were made to general hospitals of the same level. We will collect data from general hospitals in future work, and future studies will further incorporate general hospital data to verify the generalizability of the findings.

## 7 Conclusion

The DRG reform has both positive and negative effects on TCM hospitals. It has led to varying degrees of reductions in hospitalization, diagnostic, drug costs, and nursing costs in TCM hospitals. However, it also has some negative effects. First, the DRG reform has led to an upward trend in the hospitalization times of patients in the treatment group of hospitals and the phenomenon of decomposed hospitalization, which not only increases the overall burden of patients but also reflects to some extent the pressure problem of TCM hospitals under the DRG payment model. Secondly, there is a difference in the cost control effect of DRG payment reform on patients with different types of medical insurance. It indicates that TCM hospitals have patient “patient discrimination” and have not realized the payment for each type of disease and the same price for the same disease. Finally, under the incentive of the DRG payment model, TCM hospitals put economic benefits first, manifested in the reduction of labor-intensive and time-consuming Chinese medicine special techniques and therapies that have the same effect, and the increase of Western medicine therapies. In the long run, TCM hospitals will gradually become “Westernized,” threatening the uniqueness and integrity of traditional TCM services. In summary, although the DRG reform in Chinese hospitals has achieved positive results in cost control, its negative impact should not be ignored. The problem of “cost-centeredness” is particularly prominent, which poses a significant challenge to further promoting the DRG reform in TCM hospitals. Therefore, future research and policy development should focus more on exploring DRG implementation options that fit China’s national conditions, especially the characteristics of TCM, to achieve more balanced and sustainable development goals.

## Data Availability

The original contributions presented in the study are included in the article/Supplementary material, further inquiries can be directed to the corresponding author.
