# Supplementary material for: Do the diagnosis-related group payment reforms have a negative impact?—an empirical study from Western China
Source: Front Public Health. 2025 Apr 11;13:1550480. doi: 10.3389/fpubh.2025.1550480 (PMC12021814; doi:10.3389/fpubh.2025.1550480)
Supplement: Supplementary file 3 [file Table_3.docx]

Supplementary Material

# Supplementary Tables

Table Bootstrap results

|  | Observed | Bootstrap |  |  | Normal-based | |
| --- | --- | --- | --- | --- | --- | --- |
|  | coefficient | std. err. | z | P>\|z\| | [95% conf. interval] | |
| _bs_1 | -0.0076376 | 0.0004907 | -15.57 | 0.000 | -0.0085993 | -0.0066759 |
| _bs_2 | -0.1070318 | 0.0033573 | -31.88 | 0.000 | -0.113612 | -0.1004516 |
| _bs_3 | -0.1146694 | 0.0033238 | -34.5 | 0.000 | -0.1211839 | -0.108155 |

Command: sgmediation lny1, mv( RatioTCM ) iv( Treat_Post )

_bs_1: r(ind_ eff)

_bs_2: r(dir_ eff)

_bs_3: r(tot_ eff)
